# World Health Day — April 7, 2013

**Published:** 2013-04-05

World Health Day and the 50th anniversary of the World Health Organization (WHO) will be observed April 7. The focus of World Health Day this year is high blood pressure (hypertension). Although preventable, high blood pressure remains a leading risk factor for heart disease and stroke and a major cause of morbidity and mortality worldwide (1). Globally, prevalence of hypertension among adults is 40% (2), and ischemic heart disease and stroke are the first and third leading causes of premature death (3).

CDC is working to help persons control blood pressure in multiple ways, including the Million Hearts initiative. Million Hearts aims to prevent 1 million heart attacks and strokes by 2017. In addition, CDC recently released a guide to strategies to improve blood pressure control for public health practitioners (available at http://million-hearts.hhs.gov/docs/mh_smbp.pdf) and Spanish-language materials to improve health among Hispanics (available at http://millionhearts.hhs.gov/resources/toolkits.html#spanishtoolkit).

Additional information on World Health Day is available at http://www.who.int/world-health-day/en. Additional information regarding hypertension and CDC’s sodium reduction initiative is available at http://www.cdc.gov/bloodpressure, http://millionhearts.hhs.gov, and http://www.cdc.gov/salt.

## References

[1] Go AS, Mozaffarian D, Roger VL (2013). Heart disease and stroke statistics—2013 update. Circulation.

[2] Mendis S, Puska P, Norrving B (2011). Global atlas on cardiovascular disease prevention and control.

[3] Murray CJ, Vos T, Lozano R (2012). Disability-adjusted life years (DALYs) for 291 diseases and injuries in 21 regions, 1990–2010: a systematic analysis for the Global Burden of Disease Study 2010. Lancet.

